# Adaptation Aftereffects in the Perception of Crabs and Lobsters as Examples of Complex Natural Objects

**DOI:** 10.3389/fpsyg.2018.01905

**Published:** 2018-10-09

**Authors:** Antónia Reindl, Torsten Schubert, Tilo Strobach, Carola Becker, Gerhard Scholtz

**Affiliations:** ^1^Department of Psychology, Humboldt-Universität zu Berlin, Berlin, Germany; ^2^Cluster of Excellence “Image Knowledge Gestaltung – An Interdisciplinary Laboratory” Humboldt-Universität zu Berlin, Berlin, Germany; ^3^Department of Psychology, Martin-Luther-Universität Halle-Wittenberg, Halle, Germany; ^4^Department of Psychology, Medical School Hamburg, Hamburg, Germany; ^5^School of Biological Sciences, Queen’s University Belfast, Belfast, United Kingdom; ^6^Department of Biology, Humboldt-Universität zu Berlin, Berlin, Germany

**Keywords:** adaptation aftereffect, mental representation, object recognition, categorization, natural object categories

## Abstract

To recognize a familiar object, incoming perceptual information is matched against object representations in memory. Mounting evidence suggests that these representations are not stable, but adapt flexibly to recently encountered perceptual information. This is evident in the form of aftereffects, where prolonged exposure to one object (adaptor) influences perception of the next (test stimulus). So far, adaptation aftereffects have been mainly shown for human faces and simple geometric shapes, and it has been concluded that face aftereffects partially derive from shape adaptation. However, it is largely unknown whether adaptation aftereffects generalize to other categories of complex, naturalistic biological objects, and if so, whether these effects can be explained by shape adaptation. To answer these questions, we conducted three experiments in which images of crabs and lobsters were presented in two versions: as complex, naturalistic images, or reduced to their simplified geometric shapes. In Experiment 1, we found robust adaptation aftereffects for the complex versions of the images, indicating that adaptation aftereffects generalize to animate objects other than faces. Experiment 2 showed adaptation aftereffects for the simplified stimuli, replicating previous findings on geometric shapes. Experiment 3 demonstrated that adaptation to the simplified animal shapes results in aftereffects on the complex naturalistic stimuli. Comparisons between experiments revealed that aftereffects were largest in the first experiment, in which complex stimuli served as adaptor and test stimuli. Together, these experiments show that the magnitude of adaptation aftereffects depends on the complexity of the adaptor, but not on that of the test stimuli, and that shape adaptation plays a role in – but cannot entirely account for – the object aftereffects.

## Introduction

It is generally assumed that humans form mental representations and structure them into categories based on their experiences with the external world. Because of the ever-changing nature of the environment, the ability to flexibly adjust these representations to new or recently encountered information is essential for survival. Adaptation refers to the dynamic accommodation of our cognitive system to such changes. That is why looking at an object pattern for a short time typically results in a loss in sensitivity to that pattern and a bias in the appearance of other patterns (Webster, 2011), reflecting the neural strategies for optimizing perception (Thompson and Burr, 2009).

Although some theories have implicitly claimed that memory representations of object classes are stable and accurate (e.g., Bruce, 1994), mounting evidence suggests that mental representations are also subject to changes. Much of this evidence comes from adaptation studies in which morphing techniques are used to create a continuum between two extreme examples of an object class, resulting in a series of ambiguous images which gradually vary in proportion to the extremes. Usually, participants are first presented with one of the extreme stimuli as an adaptor. Afterwards, one of the ambiguous images is shown as a test stimulus and participants have to make a category decision about this stimulus. The typical finding is a contrastive effect, i.e., after the exposure to the adaptor, the test stimulus is categorized as being more dissimilar from the adaptor (the adaptation aftereffect).

Based on early findings in Gestalt psychology, it is known that current perceptual impressions of simple geometric shapes such as squares and hatches are strongly influenced by prior visual experience (Gibson, 1933, 1937; Köhler and Wallach, 1944). For example, adaptation during continuous fixation to a tilted line decreases its perceived inclination which results in an objectively vertical line appearing to be slightly tilted in the opposite direction (tilt aftereffect; see Gibson, 1933). Moreover, after seeing a screen with an ellipse (vertical ovate shape) on one side of a fixation cross, an identical figure presented symmetrically on the other side appears smaller and further away from the fixation cross (Köhler and Wallach, 1944). Also, adapting to a vertical ellipse causes a perfect circle to be perceived as slightly elliptical along the horizontal axis (Köhler and Wallach, 1944; for reviews on replications and more recent findings, see Clifford, 2002; Suzuki, 2005).

In following years, studies showed adaptation effects on different hierarchy levels of visual perception, which extended the phenomenon from adaptation effects observed in simple geometric forms to even the representations of complex visual objects consisting of combinations of smaller and larger pieces of partial or integrated shape information. Importantly, most studies focused primarily on the object category of human faces (O’Neil and Webster, 2011 and many others) while only a few studies investigated adaptation effects in other complex objects, which often represented even non-naturally, i.e., artificially created, objects such as Greebles or mixtures from, e.g., light bulbs and mushrooms (Daelli et al., 2010; see also Op de Beeck et al., 2008).

The fact that faces, due to their high social relevance for humans, represent a specific category of objects subjected to flexible adaptation, and the fact that evidence for adaptation effects in complex, natural objects (other than faces) is rather rare, has made the issue of adaptation effects in natural visual objects and their particular origins an open issue up to now. In the current study, we focused on adaptation effects in the object category of crustaceans, as this object category represents a well-defined object category allowing for the investigation of adaptation effects in complex natural objects beyond human faces and elucidates the origins for adaptation aftereffects in that object category. Before outlining the specific aims and approach of the current study, we will review former findings on adaptation aftereffects in human faces and other complex visual objects.

### Adaptation Effects in Human Faces

With regard to complex objects, human faces are perhaps the most extensively investigated object category. A number of studies have shown that the mental representation of human faces can immediately adapt to recent information (Carbon et al., 2007; Leopold et al., 2011; Strobach and Carbon, 2013). In the case of facial age, for instance, adaptation to younger or older adult faces systematically biases the subjective perception: a middle-aged face is perceived as younger after adaptation to an older face, and perceived as older after exposure to a younger face (Schweinberger et al., 2010; see also O’Neil and Webster, 2011). Prior experience also leads to face adaptation aftereffects in other category representations such as gender, ethnicity or emotional expression, both in unfamiliar faces (see Webster et al., 2004) and even in highly familiar faces (in the latter case, the effects can also be transferred to other faces, see Carbon et al., 2007). Thus, adaptation typically moves the judgment away from the adaptor – and this adaptive process can influence representations over extended periods of time (Thompson and Burr, 2009; Webster and MacLeod, 2011), meaning that the aftereffects are temporally highly robust (see Carbon and Ditye, 2011). Taken together, face aftereffects can be better explained by long-term, representational changes rather than transient, merely perceptual processes (Carbon and Ditye, 2011), suggesting that adaptation affects the representation of faces not only on the stimulus-based (retinal), but also on a higher, more abstract (memory) level.

The aforementioned studies are consistent with the assumption of norm-based theories that human faces are encoded in a multidimensional feature space centered around a prototypical norm or central tendency (Valentine, 1991, 2001). This norm reflects the average of all previously perceived faces (Thompson and Burr, 2009), and adaptation leads to changes in this feature space (Webster et al., 2004; Leopold et al., 2011). According to this model, objects are represented in a conceptual mental space along different feature dimensions (e.g., body shape). This permits an object to be classified simultaneously along multiple dimensions, rather than just by a dichotomous split along a single dimension (see Webster and MacLeod, 2011).

Importantly, existing findings also suggest that aftereffects for faces may derive in part from adaptation to shape. For example, Susilo et al. (2010) found a partial transfer of aftereffects between upright faces and T-shapes: after manipulating the height of the eyes (and thus the T-shaped spatial configuration of the eyes, nose, and mouth), and the height of the vertical bar of a T-shape, these authors concluded that almost half of the face aftereffects were due to shape-generic components. The visual system includes multidimensional spaces representing the component shapes of objects (“shape spaces”; Kayaert et al., 2005). Faces activate the visual processing stream’s several stages including a multidimensional shape space; and although upright face aftereffects originate in the face space, they consist of holistic face-specific and part-based shape-generic components which latter presumably derive from a shape space (Susilo et al., 2010). Similarly, adaptation studies on emotional expressions found facial expression aftereffects in both real faces and cartoon faces after adaptation to curved lines depicting the mouth (convex for smiling and concave for frowning; see Xu et al., 2008, 2012).

### Adaptation Effects in Complex Objects

Among the few existing studies on adaptation aftereffects in complex objects others than faces are those by Daelli et al. (2010) and Daelli (2011). The first of these studies (Daelli et al., 2010) used everyday objects, animals, and plants as endpoints for morphing and produced within-category (e.g., tree – tree, bottle – bottle) as well as between-category (e.g., cat – rabbit, light bulb – mushroom) continua. Adaptation aftereffects were found when the endpoint images served as adaptors and the ambiguous images succeeded them rapidly as test images (Daelli et al., 2010). One study (Daelli, 2011) used computer-generated Greebles (i.e., “photorealistically rendered three-dimensional objects,” Gauthier and Tarr, 1997, p. 1675) as experimental material and also demonstrated aftereffects upon adapting to endpoint images. Interestingly, in this study, increased perceptual familiarity with the object not only raised the probability of aftereffects, but also diminished the impact of low-level visual properties (such as size differences between adaptor and test images; Daelli, 2011).

Several characteristics of the objects used in earlier studies require further experimentation in order to test for a generalization of the findings to other object material. Thus, although the materials used in studies of Daelli and colleagues certainly qualify as complex stimuli, the Greebles used in Daelli (2011) are artificial and non-existent in nature; and the morphed images of everyday objects were often rather unrealistic and did not represent natural objects (Daelli et al., 2010), e.g., the morph between a light bulb and a mushroom. Furthermore, some category endpoints in Daelli et al. (2010) represent morphologically as well as genealogically unrelated objects that do not blend or co-occur in nature. As a further issue, the objects used for creating the endpoints of two different object poles in the studies of Daelli et al. (2010) were often characterized by similar geometric outer contours; for example, the head structure of mushrooms and light bulbs or trees and umbrellas which resemble objects with similar triangle-like structures. As a result, the used objects involved similar Gestalt characteristics and this makes it difficult to decide whether the mechanisms for adaptation aftereffects are merely based on shape or geometric adaptation to the outer object contour, or whether semantic characteristics of the visually presented objects contributed to the emerging adaptation aftereffects as well.

Thus, despite earlier studies which have provided valuable evidence with regard to adaptation aftereffects in complex objects, it is arguably be important to replicate these studies’ findings with further classes of objects, in particular with objects occurring in reality. In particular, it is important to test the generalizability of earlier findings for images depicting naturalistic real-life exemplars, which, if revalidated, would allow us to extend the former conclusions based on findings obtained with artificial experimental material to images of plausible models of real-life objects. In addition, it is important to disentangle the possible contributions of adaptation aftereffects occurring on the level of the geometric outer contours of the objects from contributions emerging from the processing of semantic object characteristic of the processed objects.

### The Present Study

**FIGURE 1:**
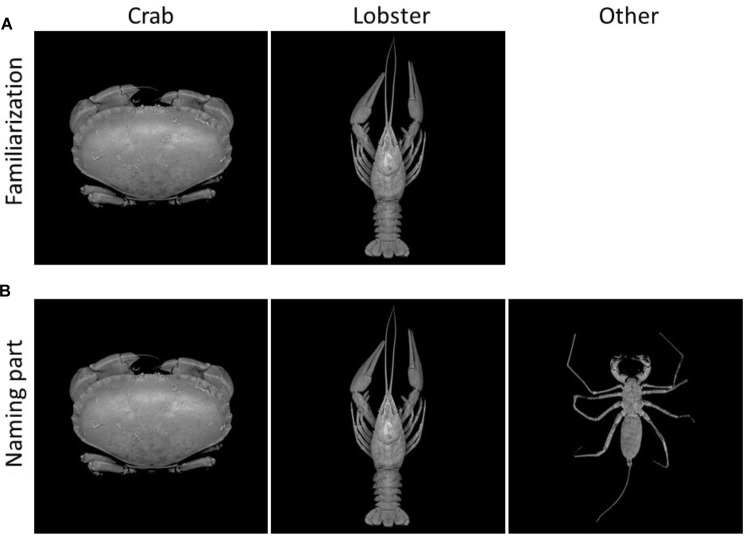
Stimulus examples from the learning phase. **(A)** Familiarization: photographs of crabs and lobster-like decapod crustaceans were presented with the respective labels of “crab” and “lobster.” **(B)** Naming: the same set of images as well as photographs from the “other” category (consisting of arachnids, as distractors) were shown without labels.

In pursuance of the aforementioned questions, we conducted three experiments, in which we investigated whether adaptation aftereffects generalize to other (compared to those used in earlier studies) complex natural, animate object categories, and if so, to what extent they can be explained by shape adaptation only. To overcome the limitations of previous investigations, the present experiments used zoological objects as examples of natural and animate categories, with category endpoints and intermediate forms that exist in reality, yet are not confounded with the high social significance of faces. Because categories or object classes on the basic level of abstraction are the most commonly used for categorization (Rosch et al., 1976), we chose examples of decapod crustaceans such as lobsters, crayfish, and crabs as basic level categories. This selection has been based on their close phylogenetic relationship, which implies a common ancestor and a comparable degree of structural complexity (see Scholtz and Richter, 1995; Shen et al., 2013). Irrespective of the concrete phylogenetic relationships, the external shape of the corpus (i.e., the carapace) of these animals allows the discrimination of two categories: the first category comprises the elongated, long-tailed decapods such as lobsters, spiny lobsters, and crayfish, which we will uniformly call “lobsters” for the sake of simplification (for an example, see **Figure 1**, center column). The other class is formed by decapods with a wide, short-tailed appearance, which are designated as “crabs” in the following (Scholtz, 2014, see **Figure 1**, left column). Both of these categories include structurally complex organisms (e.g., Keiler et al., 2015) that also have a complex outer appearance and overall shape. Thus, decapod crustaceans can be considered naturally complex stimuli from a perceptual point of view.

Lobsters and especially crabs show a great morphological diversity. Furthermore, carcinization, the evolutionary transition from long-tailed decapods to the crab shape, took place several times independently with the most prominent example of “true” crabs, Brachyura (Scholtz, 2014). This is reflected by a number of species showing intermediate forms between lobsters and crabs. Therefore, the application of a morphing technique results in zoologically plausible objects which potentially depict real animals.

**FIGURE 2:**
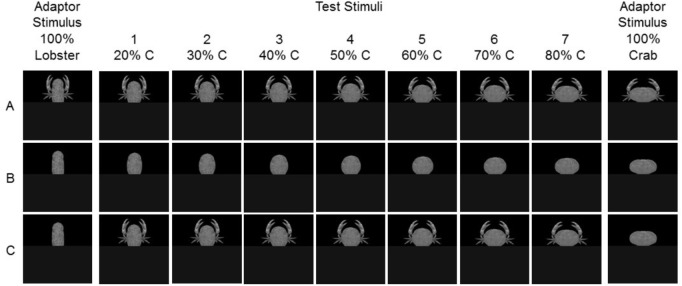
Experimental material. Rows represent the three experiments (**A**: Experiment 1, **B**: Experiment 2, **C**: Experiment 3). Columns show the two extremes 100% lobster (=0% crab, left) and 100% crab (=0% lobster, right) used as adaptor stimuli, and the seven selected morph levels used as test stimuli (percentage of C = crab indicated at top).

We used standardized photographic reproductions of certain decapod crustacean specimens in order to create a unique experimental material suited for our studies of human categorization processes. For standardization purposes and to avoid distractions from the parameters under study, we omitted some anatomical structures such as eyes and antennae. However, importantly, we added claws and legs of a mean picture of a standard crab and lobster in order to create models of a real-life image of the corresponding animals. These claws were on a midpoint of a continuum between characteristic claws and legs of an example crab and example lobster, thus not allowing a clear categorization. The resulting images (see **Figure 2**) represented illustrations resembling the transitions between the endpoints of two natural categories of crabs and lobsters including the gross outer contours of the objects added by important additional details like claws and legs. Because we were interested in the influence of continuously changing transition forms of the carapace between crabs and lobsters on human category judgments, we ordered the images linearly on a continuum changing from the pole “lobster” to the pole “crab” by systematically manipulating certain categorization-relevant features such as the shape of the carapace^1^.

This precisely controlled stimulus set allowed us to systematically investigate the influence of changes in specific features of decapods on people’s categorization judgments about crabs and lobsters. This novel material makes it therefore possible to investigate the generalizability of findings of adaptation studies in a standardized manner that was previously known mainly from the research on face perception. Using a novel and unique material in the investigation of object categorization processes requires a well-known experimental design with high general reliability. Therefore, the further methodological details are equivalent to the well-established procedures used in the case of human faces (for details, see Carbon et al., 2007; Strobach et al., 2011).

Because aftereffects can be present even a week after adaptation (see Carbon and Ditye, 2011), we chose a between-subjects design for the comparison (and thus, had a separate group of participants for each experiment) to prevent the possible biasing effects of carryover across the experiments. Since we were interested in the subjective category ratings of our artificially manipulated stimuli, we first had to ensure that all participants were aware of the objective differences between the animal groups in question. Therefore, all three experiments were preceded by a learning phase in which participants learned to accurately distinguish photographs of real crabs and real lobsters. In the subsequent test phases, we then used the adaptation task to investigate differences in the category judgments about ambiguous, morphed decapod crustaceans^2^. In Experiment 1, we tested the existence of adaptation aftereffects in complex naturalistic, morphed images of crabs and lobsters including the carapace, claws, and legs, which resulted in model illustrations of the investigated animals. In order to disentangle possible contributions by geometric form similarity between outer contours and additional visual characteristics of the presented objects we conducted Experiments 2 and 3.

In Experiment 2, we left out the claws and legs from the animals’ presentations and reduced the adaptor and test stimuli to the animals’ outer body contours resembling geometric shapes. This allowed us to test whether the emergence of adaptation aftereffects in Experiment 1 was exclusively due to geometric form adaptation oriented on the outer Gestalt of the presented carapaces or whether visual features characterizing the presented objects as members of the decapod categories, and, thus providing semantic information, contributed to the occurrence of the adaptation effects.

In Experiment 3, we tested whether adaptation to the simplified carapace shapes (i.e., as used in Experiment 2) leads to adaptation aftereffects in the complex test stimuli, which are of comparable size as the aftereffects in Experiments 1 and 2. Comparisons between the size of the resulting adaptation effects across the three experiments allowed us to determine whether the resulting adaptation effect in the current object material results merely from geometric shape adaptation due to geometric similarities of the outer contours of the animals carapaces and/or of additional contributions evoked by processing further visual information qualifying the presented objects as member of the decapod categories.

## General Methods

Because the methods of the three experiments were alike, we will give a general description of the experimental design in the following, and provide the specific details in the corresponding sections of each of the experiments.

### Participants

Healthy young adults between 18 and 35 years of age, with normal or corrected-to-normal visual acuity, and naïve to zoology, were invited from the Humboldt-Universität zu Berlin to take part in the experiments. In accordance with the Declaration of Helsinki, informed consent was acquired from all participants who were compensated for their participation (course credit or 8€/h were received as payment upon completion).

### Stimuli

Two sets of images were created for the two corresponding phases of the experiment (i.e., learning and test phase). All images were grayscale, of a size of 650 pixels × 650 pixels, and presented against a black background.

For the learning phase, 30 photographs of arthropods were equally divided into 3 different categories, namely crabs, lobsters, and others (**Figure 1**). True (brachyuran) crabs were used in the category “crab”, while long-tailed decapod crustaceans (members of the taxa Nephropidae, Astacoidea, and Munididae) formed the category “lobster.” Because of their similarity in general body shape, five species from each of the arachnid groups whip spiders (Amblypygi) and whip scorpions (Uropygi) built up the “other” category (see **Figure 1**, right column). This third category served as distractors, to ensure that participants accurately learned to recognize and differentiate the crabs and lobsters.

In order to investigate the adaptation aftereffects in the test phase, a set of standardized images was created, consisting of a series of artificially modified exemplars of crabs and lobsters. During standardization, original dorsal view photographs of real animals were first converted into grayscale images, then the body shape was subdivided into a set of modules comprising the carapace (hard dorsal shell), the pleon (abdomen or tail), the chelipeds (claws), and the pereiopods (legs). Essentially, each of the modules could be modified independently from the remaining modules. As a last step of standardization, the surface patterns of the carapaces were uniformed. A continuum with two endpoints between crabs and lobsters was formed where, because of being the sole focus of our investigation, the only manipulated feature was the *shape of the carapace*.

As illustrated in **Figure 2**, one elongated form was used for the “lobster” endpoint because of the rather universal torpedo-like carapace shape within this category. Although the forms of crab carapaces show a large variability (Ng, 1998), a transversely ovate shape was selected for the “crab” endpoint since this shape proved to be characteristic of crabs in the typological sense (Reindl et al., 2015). This assumption also resonates with the definitional criterion for crabs regarding approximately equal ratio of carapace length and width (Scholtz, 2014). In order to be able to precisely and systematically manipulate the proportion of crab and lobster traits of the carapaces for the experimental material in the test phase, WinMorph 3.01 software (DebugMode, 2006) was applied to create 100 morph images ranging from the 100% “lobster” pole to the 100% “crab” pole of the continuum. Based on being similar yet still relatively easily distinguishable from one other, seven morph levels varying in the proportion to crab (and complementary lobster) in 10% increments – namely, 20, 30, 40, 50, 60, 70, and 80% crab – were finally selected. To avoid the possibility of making category judgments based on distinctive differences between the lobsters’ and the (lack of) crabs’ tails, we covered the lower parts of the images with a dark gray rectangle. These artificially created carapaces were then used as simple contour objects resembling simple geometric shapes in Experiments 2 and 3 (**Figures 2B,C**).

Since our intention was to present not only the carapaces but rather complete animals as complex naturalistic stimuli in Experiments 1 and 3, we also added standardized claws and legs to the morphed (and partially covered) carapaces. To standardize the claws, the aforementioned morphing procedure was used across several iterations to create a neutral midpoint between crab and lobster claws, which could suit both animals. The angle of the claws was adapted in relation to the carapace to gain a more natural look (**Figures 2A,C**). In the test phases, the two endpoints, i.e., the 100% crab and the 100% lobster images, were used as adaptor stimuli, while the seven ambiguous, morphed images served as test stimuli.

### Apparatus

Participants were tested individually in a darkened room, seated approximately 60 cm from a 17″ computer screen (resolution: 1024 pixels × 768 pixels). The experimental code was written in MATLAB R2014a using Psychophysics Toolbox Version 3 for Windows.

### Design and Procedure

#### Learning Phase

The experiment started with a learning phase consisting of two successive parts. In the first, familiarization part, trials started with a black background of the screen and a light-gray fixation cross in the screen’s center for 700 ms, which was followed by 1 of the 20 photographs of crabs and lobsters. These images were presented centrally with respective category names beneath in a sequential, computer-generated random and individual order for 5000 ms per image. Participants were instructed to learn the difference between crabs and lobsters by memorizing which category each animal belonged to.

The second part of the learning phase, the naming part, consisted of trials starting with a fixation cross (as described for trials in the familiarization part), followed by showing of 1 of 30 photographs of arthropods (i.e., crabs, lobsters, and others, centrally and in randomized order), and participants were required to give individual key press responses within a 5000 ms time frame. Participants’ task in the naming part was to assign the images to 1 of the 3 categories of crabs, lobsters or other animals by pressing the keys C, M, or spacebar on a QWERTZ keyboard. Crabs and lobsters were mapped to C and M, respectively, in half of the participants, and this mapping was reversed for the other half, while the spacebar was always mapped to the third category, which served as the distractor. Feedback was given for 2000 ms after each response: the word “richtig” (German for correct) or “falsch” (German for false) and [correct category name] was written in light gray in the center of the screen. In case there was no key pressed within the 5000 ms time frame, an error message appeared pointing out the missing response and asking the participant to press 1 of the keys each time no matter what. After the 30th image, participants received overall feedback about their performance expressed in percentages, and were asked to repeat the naming part unless all their responses were correct (reaching 100% accuracy).

#### Test Phase

**FIGURE 3:**
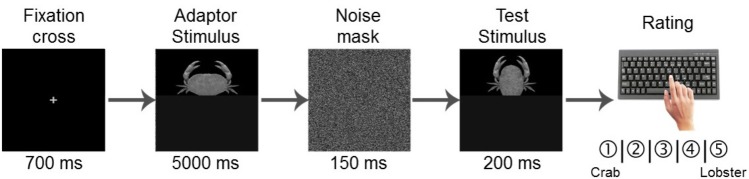
Trial structure of the test phase. First, a fixation cross appeared for 700 ms, then the adaptor stimulus was presented for 5000 ms, which was followed by a 150 ms noise mask. Then, the test image was presented for 200 ms. Finally, a blank (black) screen was shown for a maximum of 5000 ms, until the participant made a category judgment on a scale from “crab” to “lobster” by pressing one of the five possible keys.

In the test phase, the experimental material consisted of the adaptor stimuli (both, 100% crab and 100% lobster endpoints of the morphing continuum) and the test stimuli (the 7 ambiguous, morphed images ranging from 20 to 80% crab, **Figure 2A**), which were paired (1 adaptor to 1 test image) to form a total of 14 pairs. Similar to the learning phase, the images were presented centrally and separately in semi-randomized order; this meant that the sequence of these pairs was incidental within as well as across participants. Each individual trial was then structured in the following manner (**Figure 3**): first, the fixation screen appeared for 700 ms. Second, one of the 2 adaptor stimuli was presented for 5000 ms. Third, and in order to prevent the possible bias of a retinal afterimage, one of the 2 Fourier phase-randomized versions of the adaptors was randomly shown for 150 ms as a noise mask. Fourth, one of the test stimuli was presented for 200 ms. Fifth and last, a blank (black) screen was shown until a key press, not exceeding 5000 ms. Participants’ task was to thoroughly observe the first, adaptor image and then categorize the second, test image (after its presentation, during the 5000 ms blank screen) on a scale from “1” to “5” by pressing 1 of the corresponding keys C, V, B, N, and M. For half of the participants, “1” to “5” was mapped to crab vs. lobster, respectively, while the remaining participants received a reversed mapping. The mapping order across the learning and test phases was kept the same for each participant.

The test phase comprised a total of 4 blocks of 56 trials each in which the 14 individual stimulus pairs were presented 4 times. Each block started with 6 warm-up trials and was followed by a short, participant-paced break.

### Analysis

During data processing, the reversed mapping scale was recoded to “1” = crab and “5” = lobster. Category ratings were measured as dependent variables after being averaged for each (morphed) test image across the blocks. A 2 × 7 repeated measures analysis of variance (ANOVA) was calculated with the two within-subjects factors Adaptor Stimulus (100% crab and 100% lobster) and Test Stimulus (Test Stimulus 1 of 20% crab, Test Stimulus 2 of 30% crab […] Test Stimulus 7 of 80% crab) on participants’ judgments. If necessary, we applied separate pairwise *t*-tests for testing possible differences between mean values; with Bonferroni-correction if multiple comparisons were conducted. Warm-up trials, along with responses faster than 200 ms and missing responses (i.e., responses exceeding the 5000 ms time limit) were excluded from the analysis.

## Experiment 1

Experiment 1 was based on previous findings on complex object classes, both natural (human faces) and artificial (used by Daelli et al., 2010; Daelli, 2011). It tested the existence of adaptation aftereffects in alternative categories of animate, complex natural object representations, using naturalistic, composite images of decapods. Since previous studies could show adaptation aftereffects for complex stimuli (i.e., Daelli, 2011), we hypothesized to find measurable aftereffects using the described naturalistic material of morphed models of crabs and lobsters.

### Methods

#### Participants

A total of 32 volunteers (21 females; mean age = 28.1 years, *SD* = 4.2 years) took part in the experiment, of whom 6 were left-handed. The data of two participants were excluded from the analysis, due to an extremely strong bias toward the rating “crab” (more than 96% of valid responses).

#### Stimuli

Composite, naturalistic images consisting of the morphing continuum of standardized carapaces, and completed with morphed claws and legs were used. The 2 endpoints of the continuum (i.e., the 100% crab and the 100% lobster) served as adaptor, and the 7 morphed, ambiguous variants as test stimuli.

### Results

**FIGURE 4:**
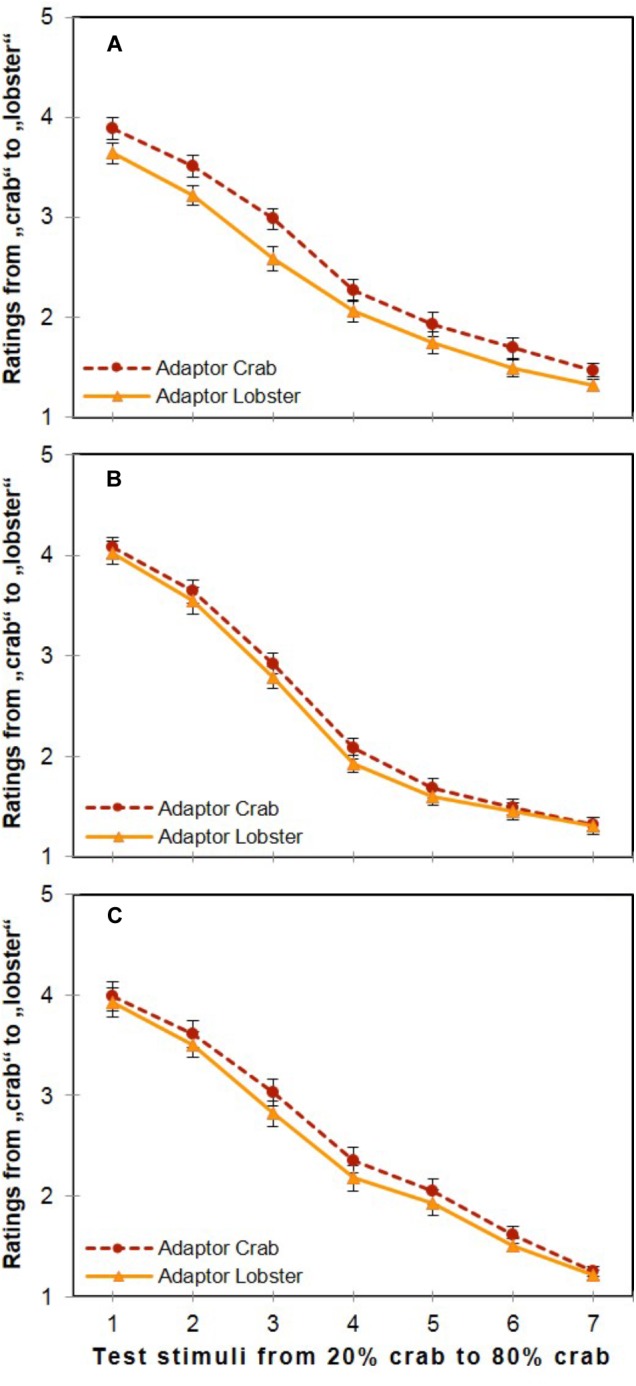
Overall ratings in the three experiments. Effects of the two adaptor stimuli (100% crab and 100% lobster) and the seven morph levels on category ratings ranging from “crab” to “lobster” in the three experiments (**A**: Experiment 1, **B**: Experiment 2, **C**: Experiment 3; error bars show the standard error of the mean).

Participants’ category ratings regarding the test stimuli reflected a strong influence of both the two adaptor stimuli and the seven different variations of the test stimuli themselves. As illustrated in **Figure 4A**, a significant main effect was shown for both Adaptor Stimulus [*F*(1,29) = 36.730, *p* < 0.001, $\eta_{p}^{2}$ = 0.559], and for Test Stimulus [*F*(6,174) = 192.828, *p* < 0.001, $\eta_{p}^{2}$ = 0.869], which also interacted significantly [*F*(6,174) = 2.263, *p* = 0.040, $\eta_{p}^{2}$ = 0.072]. The main effect of Adaptor Stimulus indicated the presence of strong adaptation aftereffects, since the mean value of ratings after seeing the 100% crab adaptor (*M* = 2.53, *SD* = 1.20, *SE* = 0.079) was significantly larger (i.e., closer to “lobster”) than after the 100% lobster adaptor (*M* = 2.29, *SD* = 1.22, *SE* = 0.076) on the unified mapping scale of “1” = crab and “5” = lobster.

With respect to the main effect of Test Stimulus, the ratings for each test image were averaged across the two adaptors, and Bonferroni-adjusted *post hoc* comparisons showed significant differences between all possible pairs (Δ*M*s ≥ 0.245, *SE*s ≤ 0.137, *p*s ≤ 0.001). This result indicates that the participants are able to distinguish between the seven different test images of varying morph levels.

In the case of the two factors’ interaction, the ratings after seeing the 100% crab adaptor were significantly larger than the ratings after the 100% lobster adaptor at all seven test images [all *t*s(29) ≥ 3.089, *p*s ≤ 0.004], the effect sizes of the adaptation aftereffects were the largest for two highly ambiguous test images at intermediate positions of the morphing continuum. That is, adaptation aftereffects were the largest at Test Stimulus 3, the subjective midpoint of the continuum (based on the participants’ ratings, see **Figure 4A**) containing 40% crab [Δ*M* = 0.39; *F*(1,29) = 25.489, *p* < 0.001, $\eta_{p}^{2}$ = 0.468], and Test Stimulus 4, the objective midpoint of the continuum containing 50% crab [Δ*M* = 0.31; *F*(1,29) = 19.980, *p* < 0.001, $\eta_{p}^{2}$ = 0.408]. The adaptation aftereffects were smaller for the remaining five test stimuli at other positions [Δ*M*s ≤ 0.29; *F*s(1,29) ≤ 14.482, *p*s ≥ 0.001, $\eta_{p}^{2}$s ≤ 0.333]. That is, the influence of the adaptor stimuli was weaker at the endpoints of the morphing continuum compared to the midpoints, perhaps because here the exemplars showed the largest contribution of the features defining the corresponding category.

### Discussion

The category judgments regarding the test stimuli were found to be strongly influenced both by the presented adaptor stimulus and by the test stimulus. This demonstrates the existence of adaptation aftereffects in animate, complex natural object categories other than human faces – as well as the participants’ ability to differentiate the various morph levels of the test stimuli. The findings are consistent with earlier findings of Daelli et al. (2010) and Daelli (2011) who also used complex non-face stimuli and found valid adaptation aftereffects. However, the current stimulus material was derived from morphs of natural object categories, i.e., crabs and lobsters, which differ from the material in earlier studies. The material in these earlier studies emerged from rather artificial object representations, e.g., a morph between a light bulb and a mushroom, or between two types of Greebles. Taken together the findings of the current study, along with earlier studies, provide sufficient evidence suggesting that adaptation aftereffects can be found in a large range of complex visual objects with more or less naturalistic origins.

It is important to note that the current models of crabs and lobster models allow for further disentangling the contribution of different sources of information conveyed by the presented models for the adaptation aftereffects. Thus, the current animal models consisted of distinguishing features, such as the carapace shapes, and, in addition, of further details, such as claws and legs, which were commonly mounted to the carapaces of the crabs and lobsters in order to make the current object representations more plausible members of decapod categories.

Since in Experiment 1, the shape of the carapaces was the only differentiating feature allowing for a decision about the category, the current results could be explained by an account assuming simple shape adaptation as the main source for the current findings. According to that assumption, the observed aftereffects could be due to shape adaptation, merely to the varying part of the stimuli (i.e., the carapaces’ shape, outer contour), instead of adaptation to the stimuli as a whole (implying the mental representations of decapods including the representation of additional information like claws, legs, etc.). To investigate the possibility of this explanation, and to specify the potential contribution of shape adaptation and of further information qualifying the presented objects as more complex naturalistic objects of the decapod categories, we conducted Experiments 2 and 3.

## Experiment 2

Based on their resemblance to simple contour (ovate and circular) shapes, the carapaces generated for Experiment 1 could be considered as simple geometric stimuli and the resulting adaptation aftereffect as resulting from pure geometric form adaptation. It has been known since earlier studies of Gibson (1933, 1937) and Köhler and Wallach (1944) that strong adaptation effects can be evoked by adapting to ovate and circular shapes as geometric forms. Thus, the adaptation aftereffects observed in Experiment 1 could be due to adaptation only to the corresponding geometric shape of the carapace, and not to the mental representations of complex naturalistic decapod objects.

In order to test whether adaptation aftereffects could have been elicited merely by adapting to the geometric outer contours of the carapaces of the presented animals we carried out Experiment 2, in which we presented only the carapaces of the crabs and lobsters and skipped the claws and legs of the animals. As can be seen in **Figure 2**, the creation of the corresponding material leads to object representations of ovate and elongated shapes with the same texture and color characteristics as the crab and lobster representations in Experiment 1, however, without additional object details like claws and legs; the latter would qualify the objects as a member of the decapod categories.

In Experiment 2, participants were enrolled in the same learning phase as in Experiment 1, in which they learned to distinguish between characteristic examples of crabs and lobsters. In the subsequent test phase participants judged the presented objects (i.e., the morphed carapaces of crabs and lobsters without claws and legs) as characteristic examples of crabs or lobsters after the perception of the adaptor stimuli on a 5-point judgment scale (see Experiment 1). If Experiment 2 leads to adaptation aftereffects of the same size as in Experiment 1, then the emergence of adaptation aftereffects can be explained by pure simple shape adaptation at the level of the presented carapaces. However, if the processing of additional object characteristics determining the presented visual objects as members of the decapod categories crabs and lobsters contributes additionally to the emergence of adaptation aftereffects, then the size of the adaptation aftereffect in Experiment 2 should be smaller than in Experiment 1.

### Methods

#### Participants

In order to determine the adequate sample size for Experiment 2, we conducted a power analysis by simulation, based on the results from Experiment 1. We simulated 10000 new data sets of 20 participants each, by creating new mean ratings for each combination of adaptor and test stimulus. The mean ratings for the lobster adaptor were drawn from normal distributions with means equal to the observed means obtained in Experiment 1, and standard deviations equal to the observed standard deviation. The mean ratings for the crab adaptor were drawn from the same normal distributions, but with means 0.24 of a point greater than for the lobster adaptor (i.e., by adding the observed main effect of crab adaptor from Experiment 1). For each simulated data set, we recorded whether the same ANOVA procedure as described for the other experiments detected a significant main effect of adaptor stimulus at α = 0.05.

We found that just over 93% of the simulated data sets yielded a significant test of our hypothesis. Thus, at a sample size of 20, we estimated that the power of Experiment 2 to detect the same main effect of adaptor stimulus as in Experiment 1 would be approximately 0.93. Accordingly, twenty participants (18 females; mean age = 26.4 years, *SD* = 5.6 years) were recruited, of whom 3 were left-handed. None of the participants’ data was excluded from the analysis since the distribution of the individuals’ category ratings did not indicate any strong bias.

#### Stimuli

Similar to Experiment 1, Experiment 2 comprised the 2 successive phases of the learning phase and the test phase. The 30 images of the learning phase were identical to the ones used in Experiment 1 (**Figure 1**). The test phase, however, consisted of simple contour images of the carapaces used in Experiment 1. In other words, we presented the images of artificially created decapod carapaces used in Experiment 1 with the distinction that extremities (claws and legs) were skipped from the adaptor stimuli and from the test stimuli (**Figure 2B**).

### Results

Adaptor Stimulus showed a significant main effect [*F*(1,19) = 7.112, *p* = 0.015, $\eta_{p}^{2}$ = 0.272]; the mean value of ratings after seeing the 100% crab adaptor was significantly larger than after the 100% lobster adaptor (*M* = 2.46, *SD* = 1.22, *SE* = 0.053 and *M* = 2.38, *SD* = 1.21, *SE* = 0.054, respectively).

Another main effect was found for Test Stimulus, [*F*(6,114) = 155.304, *p* < 0.001, $\eta_{p}^{2}$ = 0.891], where the differences were significant between all possible pairs of the Bonferroni-corrected *post hoc* comparisons (Δ*M*s ≥ 0.156, *SE*s ≤ 0.172, *p*s ≤ 0.022). However, the interaction of these two factors was not significant [*F*(6,114) = 0.933, *p* = 0.475, $\eta_{p}^{2}$ = 0.047], as illustrated in **Figure 4B**.

All in all, these findings indicate the presence of adaptation aftereffects with the stimulus material based on the pure carapace representations of Experiment 1 and this indicates that simple geometric form adaptation contributes to the emerging size of the adaptation aftereffects in the object material of Experiment 2.

In a subsequent analysis, we compared the size of the adaptation aftereffects in Experiments 1 and 2, in order to test whether they are of similar size or not. For that purpose, we ran an ANOVA, with the between-subjects factor Experiment (1 vs. 2) and the within-subjects factors Adaptor and Test stimulus on the rating values of the subjects from Experiments 1 and 2. The analysis showed that the adaptation aftereffect was smaller in Experiment 2 (*M* = 0.08, *SD* = 0.136, *SE* = 0.038) than in Experiment 1 (*M* = 0.24, *SD* = 0.217, *SE* = 0.031), which is indicated by the significant interaction of Experiment and Adaptor Stimulus [*F*(1,50) = 7.288, *p* < 0.005, $\eta_{p}^{2}$ = 0.127]. Consequently, processing of further object characteristics (claws and legs) determining the presented visual objects as members of the semantic decapod categories crabs and lobsters (Experiment 1), additionally contributes to the emergence of adaptation aftereffects to pure geometric form adaptation (Experiment 2). The comparison of Experiments 1 and 2 also showed significant main effects of Adaptor Stimulus [*F*(1,50) = 32.331, *p* < 0.001, $\eta_{p}^{2}$ = 0.393], and Test Stimulus, [*F*(6,300) = 287.459, *p* < 0.001, $\eta_{p}^{2}$ = 0.852], as well as a significant interaction of Test Stimulus and Experiment [*F*(6,300) = 4.565, *p* < 0.001, $\eta_{p}^{2}$ = 0.084].

### Discussion

In Experiment 2, we found adaptation aftereffects also in the isolated carapaces resembling simple geometric shapes. Again, the main effect of Test stimulus indicated that participants could effectively differentiate between the seven morph levels of the carapace shapes. This indicates that adaptation to the outer contours of the presented carapace stimuli can lead to adaptation aftereffects in the current stimulus material, which represents a rudimentary shape representation of the complex visual objects of crab and lobster stimuli as presented in Experiment 1.

However, the results of a subsequent cross-experiment comparison showed that the size of the adaptation effect in Experiment 2 is far smaller than that in Experiment 1. This indicated that the additional presentation of claws and legs in Experiment 1 provided additional information causing larger adaptation aftereffects in the Experiment 2 set of crab and lobster models than did the pure geometric shape representation of the presented carapace stimuli only. This enlarged the adaptation aftereffects in the current material to great extent.

Importantly, the amount of the increase of the adaptation aftereffect in Experiment 1 compared to Experiment 2 was similar across all seven morph stages of the Test stimuli as had been confirmed by the non-significant interaction between Adaptor, Test stimulus, and Experiment. This is consistent with the assumption that the additional information provided by the claws and legs affected the category judgments in equal manner across the morphing stages and rules out a gradual influence of the visual detail information provided by the claws and legs. This in turn, is consistent with the fact that the claws and legs did not provide any discriminative information about the presented carapaces but allowed only in general to relate the presented objects to the crustacean decapod categories.

## Experiment 3

Experiment 3 was performed to further elucidate the impact of the geometric shape information provided by the carapace, and the potential impact of further visual object characteristics provided by claw and leg information for the resulting adaptation aftereffects. For that purpose, in the adaptation phase of Experiment 3, we presented, as adaptation stimuli, only presentations of the carapaces of the crab and lobster models, i.e., without the claws and legs, as in Experiment 2. However, differently to Experiment 2, we presented the complex test stimuli of Experiment 1, which included the carapace, the claws, and the legs (**Figure 2C**). In this way, Experiment 3 adapted procedures from investigations on face adaptation effects partially deriving from shape adaptation (see Xu et al., 2008, 2012; Susilo et al., 2010). If Experiment 3 leads to adaptation aftereffects of the same size as in Experiment 1, then the emergence of these effects can be explained by an assumption that pure geometric shape adaptation at the level of the presented carapaces can evoke adaptation effects on the level of the decapod categories, if the test stimuli provide visual information that additionally enriches the presented objects on the semantic, i.e., category, level. However, if the processing of additional object characteristics (i.e., claws and legs) in the adaptation phase is inevitably necessary to evoke the full adaptation aftereffects, then the size of the aftereffect in Experiment 3 should be smaller than the ones in Experiment 1.

With the present set of stimuli, we could also test an alternative explanation of the findings in Experiments 1 and 2. According to that explanation, the emergence of the adaptation aftereffects could have resulted from structural superficial form similarity between the adaptation and the test stimuli in these experiments. Note that in Experiments 1 and 2, both the adaptation stimuli and the test stimuli, were of similar outer contours (with the material in Experiment 1 including the details of claws and legs, and the material in Experiment 2 lacking that additional information). If the mere structural similarity between adaptation and test stimuli would have been decisive for the emergence adaptation aftereffects, we would expect adaptation effects in Experiments 1 and 2 but not in the present Experiment 3. If on the contrary superficial form similarity is not decisive for the emergence of adaptation aftereffects, then we should find a significant adaptation aftereffect in Experiment 3.

### Methods

#### Participants

Twenty-eight persons (22 females; mean age = 27.3 years, *SD* = 6.2 years; 5 left-handed) volunteered to take part in the experiment.

#### Stimuli

Stimuli in the learning phase were identical to those in Experiments 1 and 2 (**Figure 1**). In the following test phase, the 2 endpoints of the simple contour, geometric shapes used in Experiment 2 (i.e., the 100% crab and the 100% lobster carapaces) served as adaptor stimuli, while the 7 morphed images of complete animals (carapaces with extremities) from Experiment 1 were used as test stimuli (**Figure 2C**).

### Results

Both the two factors’ main effects and their interaction turned out to be significant. For the main effect of the factor Adaptor Stimulus [*F*(1,27) = 25.781, *p* < 0.001, $\eta_{p}^{2}$ = 0.488], the mean value of ratings after seeing the 100% crab adaptor (*M* = 2.56, *SD* = 1.25, *SE* = 0.099) was significantly larger than after the 100% lobster adaptor (*M* = 2.44, *SD* = 1.23, *SE* = 0.093). For the main effect of Test Stimulus [*F*(6,162) = 223.499, *p* < 0.001, $\eta_{p}^{2}$ = 0.892], the Bonferroni-adjusted comparisons revealed significant differences between all possible pairs (Δ*M*s ≥ 0.278, *SE*s ≤ 0.147, *p*s ≤ 0.001).

With respect to the two factors’ interaction [*F*(6,162) = 2.479, *p* = 0.025, $\eta_{p}^{2}$ = 0.084], the Bonferroni-adjusted multiple *t*-tests showed that the ratings after the crab adaptor were significantly larger than after the lobster adaptor at the five intermediate positions of the morphing continuum (i.e., at Test Stimulus 2 containing 30% crab, at Test Stimulus 3 of 40% crab, at Test Stimulus 4 of 50% crab, at Test Stimulus 5 of 60% crab, and at Test Stimulus 6 of 70% crab; all *t*s(27) ≥ 2.225, all *p*s ≤ 0.035): the largest effects were observed at the two most ambiguous images, at Test Stimulus 3 of 40% crab, which was the subjective midpoint based on the participants’ category ratings (Δ*M* = 0.210, *SE* = 0.046, *p* < 0.001), and at Test Stimulus 4 of 50% crab, which was the objective midpoint (Δ*M* = 0.179, *SE* = 0.050, *p* = 0.001). The effects were smaller at the remaining three intermediate positions: Δ*M* = 0.107 (*SE* = 0.048, *p* = 0.035) at Test Stimulus 2 of 30% crab, Δ*M* = 0.110 (*SE* = 0.033, *p* = 0.002) at Test Stimulus 5 of 60% crab, and Δ*M* = 0.106 (*SE* = 0.036, *p* = 0.007) at Test Stimulus 6 of 70% crab. Differences of ratings after crab and lobster adaptors were not significant at the peripheral positions of the continuum, i.e., neither at Test Stimulus 1 of 20% crab (Δ*M* = 0.061, *SE* = 0.047, *p* = 0.207), nor at Test Stimulus 7 of 80% crab (Δ*M* = 0.038, *SE* = 0.031, *p* = 0.230; see **Figure 4C**).

### Discussion

Experiment 3 showed adaptation aftereffects in situations, in which participants adapted to the pure carapace shape adaptor stimuli (i.e., the rudimentary ovate and elongated shapes of Experiment 2) and transferred to the complex, naturalistic stimuli including the carapace and additional visual information qualifying the presented objects as members of the lobster and crab categories. This rules out that structural superficial form similarity between the adaptation and the test stimuli is the decisive precondition for the emergence of adaptation aftereffects with the current stimulus material.

Furthermore, and most importantly, the findings of Experiment 3 allow for an elaborated estimation of the impact of the pure shape information and of the impact of the information enriching details like claws and legs during the adaptation phase and the transfer to the testing phase. To elucidate the particular impact of these components, we conducted a detailed comparison between the sizes of the adaptation aftereffects across the three experiments, which will be reported next.

## Comparison of Experiments 1, 2, and 3

In the subsequent comparison of the adaptation aftereffects in the three experiments, we tested several assumptions about the potential impact of the (carapace) shape information and of the additional impact by further visual information qualifying the presented objects on a semantic level as members of the crustacean decapods. First of all, we tested whether the adaptation aftereffects in complex vs. simple object representations are exclusively due to geometric shape adaptation. If that were the case, then we would expect the strongest effects in the case of the simple contour stimuli of Experiment 2. Weaker effects would have been predicted for the mixed conditions of Experiment 3, where the carapaces, shown as simple shapes, were the adaptors, and the complex naturalistic animal models (carapaces with claws and legs) were presented as the test images, followed by the adaptation effects in Experiment 1 (complete adaptors and test images). The reason behind this prediction is that the only manipulated and, thus, task-relevant feature was the shape of the carapace, while the features making the stimuli naturalistic and thus complex were completely task-irrelevant, assuming to be distracting and thus diminishing the adaptation aftereffects (cf. Awh and Pashler, 2000; Brisson and Jolicśur, 2007).

On the contrary, if complex naturalistic object representations including the information about the carapace shape and detailed object information qualifying the objects on a semantic level as members of the crustacean categories would be decisive for evoking the full adaptation mechanism, then we would expect the opposite pattern of results. Namely, adaptation aftereffects should be strongest in the case of the complex, naturalistic stimuli in Experiment 1, weaker in the mixed conditions (simple shape adaptors and complex, naturalistic test images) in Experiment 3, and weakest for the simple geometric images in Experiment 2. This hypothesis would be consistent with the assumption that the extremities of the animals and their positions provide additional cues to elicit adaptation aftereffects.

As a further possibility, we could expect to replicate the results of Susilo et al. (2010) who found significantly smaller aftereffects in faces following adaptation to T-shapes compared to both congruent conditions (i.e., face adaptors with face test images and shape adaptors with shape test images, without any statistical difference between these two conditions).

### Analysis

#### Comparison of Experiments

As a first step, we calculated the numerical values of the aftereffects separately in all three experiments: since adaptation aftereffects reflect the adaptor stimuli’s influence on the participants’ ratings at each separate morph level, the ratings given after seeing the 100% lobster adaptor were subtracted from the ratings given after the 100% crab adaptor (at each morph level, and for each participant, respectively). The resulting difference values represent the size of the influence with which the adaptor stimulus (lobster and crab) affected the category ratings of participants. The larger the difference value, the larger the amount of the adaptation effect in participants’ category ratings, since the presentation of the crab adaptor should lead to larger judgment values (i.e., to a more lobster-like rating on the unified mapping scale from “1” = crab to “5” = lobster) and vice versa for the case of the lobster adaptor. Subsequently, we calculated a two-way mixed-measures ANOVA with the within-subjects factor Test Stimulus (from 20 to 80% crab) and the between-subjects factor Experiment on the numerical values of the adaptation aftereffects (i.e., the described difference values).

### Results and Discussion

#### Comparison of the Adaptation Aftereffects Across the Three Experiments

We found significant effects of both factors, Experiment and Test stimulus. The significant effect of the factor, Experiment [*F*(2,75) = 6.598, *p* = 0.002, $\eta_{p}^{2}$ = 0.150], indicates different sizes of the adaptation effect in the three experiments. *Post hoc* comparisons with separate *t*-tests showed that the adaptation effect in Experiment 1 (*M* = 0.24, *SD* = 0.031) was significantly larger than in both, Experiment 2 (*M* = 0.081, *SD* = 0.136, *SE* = 0.038; *p* = 0.005) and Experiment 3 (*M* = 0.116, *SD* = 0.120, *SE* = 0.032, *p* = 0.018). The difference of the adaptation aftereffects between Experiment 2 and 3 was not significant (*p* > 0.20).

In addition, the within-subjects factor Test Stimulus showed a significant effect [*F*(6,450) = 3.902, *p* = 0.001, $\eta_{p}^{2}$ = 0.049] on the size of the adaptation effects. Subsequent Bonferroni-adjusted multiple *t*-tests revealed that the adaptation aftereffects at an intermediate position, i.e., at the subjective midpoint of the morphing continuum (i.e., Test Stimulus 3, the image containing 40% crab) were significantly larger than at both of the peripheral positions [i.e., Test Stimulus 1 of 20% and Test Stimulus 7 of 80% crab; *t*s(77) ≥ 3.465, *p*s ≤ 0.001]. Furthermore, the adaptation aftereffects at the objective midpoint of the morphing continuum (Test Stimulus 4 containing 50% crab) were also significantly larger than at Test Stimulus 7, the image of 80% crab [*t*(77) = 3.123, *p* = 0.003]. Thus, the main effect of Test Stimulus showed that the elicited adaptation aftereffects at the two intermediate positions of 40 and 50% crab (the subjective and the objective midpoints) were significantly larger than at the peripheral positions (or at least one of them, the 80% crab) across all experiments. This is consistent with the assumption that the most ambiguous images (at the center of the morphing continuum) are much more flexible and subject to influence (and presumably also much harder to categorize) than the ones at the two peripheral positions being thus the closest to the respective norms (see Webster et al., 2004; Xu et al., 2008, 2012; Daelli et al., 2010). The lack of an interaction between Test Stimulus and Experiment [(*F*(12,450) = 0.671, *p* = 0.780, $\eta_{p}^{2}$ = 0.018], indicates that the observed pattern of different sizes of the adaptation effects across the different morph stages of the seven test stimuli was similar across the three experiments.

#### Contribution of Shape Adaptation to Object Aftereffects

In order to determine the specific contribution of shape adaptation to the adaptation aftereffects in the current material, we conceptually replicated the analysis of Susilo et al. (2010) by computing the proportion of the numerical values of the adaptation aftereffects in Experiment 3 and Experiment 1 [(Δ Adaptor Stimulus_Experiment 3_/Δ Adaptor Stimulus_Experiment 1_) × 100]. This comparison gives a good approximation of the size of shape adaptation because participants adapted in Experiment 1 to the complex, naturalistic adaptor stimuli and in Experiment 3 to the shape (carapace) stimuli, while the transfer occurred to the same type of complex naturalistic stimuli in both experiments.

The analysis revealed that about 50% of the adaptation aftereffects in Experiment 1 (*M* = 0.24) could be explained by shape adaptation from Experiment 3 (*M* = 0.12). This implies that almost half of the adaptation aftereffect is not object-specific but presumably derived from a multidimensional shape space – which resonates with previous findings (see Watson and Clifford, 2003, 2006; Susilo et al., 2010).

#### Comparison of the Reaction Times Across Experiments

To assess whether there were any possible differences in task difficulty between the experiments, we analyzed the reaction times in the three experiments by conducting a 2 × 7 × 3 mixed-measures ANOVA with the within-subjects factors Adaptor Stimulus and Test Stimulus, and the between-subjects factor Experiment. If there were any differences in difficulty between the three experiments, then this could have confounded the ratings of participants in the different conditions of the presented stimuli. However, we did not find a significant effect of the factor Experiment on reaction times [*F*(2,75) = 0.244, *p* = 0.784, $\eta_{p}^{2}$ = 0.006]: the mean values were *M* = 828 ms (*SD* = 165 ms) in Experiment 1, *M* = 869 ms (*SD* = 231 ms) in Experiment 2, and *M* = 833 ms (*SD* = 162 ms) in Experiment 3. As we did not find any significant interaction effects between Experiment and any of the other factors on reactions times, either, we conclude that the difficulty levels were comparable in the three experiments and a theoretically potential influence of the difficulty level on the rating values can be ruled out (see **Table 1**).

**Table 1:** Mean reaction times (in ms) across the three experiments.

Adaptor	Test Stimulus						
	
	1	2	3	4	5	6	7
**Experiment 1**							
Crab	886(231)	907(221)	928(168)	877(216)	747(172)	786(189)	793(211)
Lobster	968(257)	970(225)	917(229)	787(178)	770(221)	743(189)	732(191)
**Experiment 2**							
Crab	921(267)	938(281)	1026(318)	944(345)	826(323)	772(220)	717(210)
Lobster	945(299)	986(248)	1044(321)	860(323)	752(263)	733(259)	705(207)
**Experiment 3**							
Crab	838(182)	850(189)	962(253)	887(191)	774(195)	779(211)	739(189)
Lobster	899(209)	922(210)	956(201)	813(200)	792(213)	750(199)	707(189)
**Overall**							
Crab	877(224)	894(227)	965(244)	898(246)	777(226)	780(202)	754(203)
Lobster	937(251)	957(225)	964(249)	815(229)	773(227)	743(210)	716(192)


*Standard Deviations appear in parentheses below means.*

The analysis showed no further main effect of the Adaptor Stimulus on reaction times, either [*F*(1,75) = 1.432, *p* = 0.235, $\eta_{p}^{2}$ = 0.019]. The only significant main effect was found for Test Stimulus [*F*(2.845,213.373) = 37.223, *p* < 0.001, $\eta_{p}^{2}$ = 0.332]. The latter is caused by longer reaction times on the Test Stimuli 1 (*M* = 907 ms), 2 (*M* = 926 ms), 3 (*M* = 965 ms), and 4 (*M* = 856 ms) compared to the reaction times at the Test Stimuli 5, 6, and 7 (*M* = 775, 761, 735 ms, respectively; all *p*s < 0.001; multiple *t*-tests with Bonferroni -corrections). The significant interaction of Adaptor and Test Stimulus [*F*(5.207,390.515) = 9.827, *p* < 0.001, $\eta_{p}^{2}$ = 0.116] reflects the fact that reaction times after seeing the 100% crab adaptor were significantly smaller than after the 100% lobster adaptor at the first two test images (*p*s ≤ 0.003), and significantly larger at Test Stimulus 4, 6, and 7 (*p*s ≤ 0.026). This pattern plausibly reflects the mechanisms of adaptation: the images that are more similar to the given adaptor are more difficult to judge, i.e., the categorization of images on the (subjective) “crab” side of the continuum takes more time after being adapted to the 100% crab, while the same is true for the “lobster” side after the lobster adaptor.

In sum, the analysis of the reaction times showed no signs for differences in the task difficulty among the experiments, and the pattern of results suggests that the images can easier be processed on the intermediate positions of the morphing continuum as compared to the outer positions.

## General Discussion

The aim of the present study was to investigate whether adaptation aftereffects extend to further categories of animate, complex biological object representations beyond human faces, and if so, whether and to what degree these effects derive from shape adaptation. For this purpose, we took standardized images of crabs and lobsters (i.e., decapod crustaceans) as two general classes of animate, complex natural objects. Morphing techniques from face research were then used to systematically manipulate the carapaces (dorsal shells) on an artificial yet zoologically plausible continuum depicting the evolutionary transition from a lobster-like shape to the crab. We created two stimulus sets of increasing complexity: one comprising the mere carapaces as simple contour images resembling simple geometric shapes, and another with extremities such as claws and legs added to the carapaces, and thus consisting of complex naturalistic images. The lower parts of the stimuli were always covered in order to eliminate the possible bias introduced by the tail which is either present or absent and would otherwise have a major impact on the decision of laypersons. As prototypical exemplars representing the norms of their classes, the endpoints of the continua served as adaptors, and the morphed images depicting intermediate transition levels were used as test stimuli to measure the adaptor-dependent differences in category ratings for the ambiguous objects.

In Experiment 1, we used the complex stimulus set for both the adaptors and the test images and found robust adaptation aftereffects, indicating that adaptation and the resulting aftereffects generalize to representations of alternative animate, complex natural object categories. In Experiment 2 with the simple stimulus set (omitting the claws and legs), we found aftereffects in the mere carapaces resembling simple geometric objects and thus successfully replicated findings of earlier shape adaptation studies. In Experiment 3, where the mere carapaces served as adaptors and the complex naturalistic stimuli (including claws and legs) as test images, our results demonstrated the transferability of aftereffects from simple geometric shapes to complex, naturalistic object representations.^3^

The direct comparison of the adaptation aftereffects in the three experiments provided important findings: first, since the aftereffects in Experiment 1 were significantly larger than in both Experiments 2 and 3, the magnitude of adaptation aftereffects seems to depend on the complexity of the adaptor, regardless of both the adaptor – test image congruence and the complexity of the test stimulus. Second, since the aftereffects were the largest (and the reaction times the longest) at the subjective and objective midpoints of the morphing continuum as compared to the peripheral morph levels, it appears that the degree of the representations’ flexibility is a function of their distance from their norms. In other words, the most ambiguous images are much more prone to influence and harder to categorize than the ones which are close to the respective norms. Third, since almost half of the size of the adaptation aftereffects seems to derive from shape adaptation, our results resonate with previous studies (see Watson and Clifford, 2003, 2006; Susilo et al., 2010), and support the assumption not only of a multidimensional shape space (cf. Pasupathy and Connor, 2001; Kayaert et al., 2005), but also of a multidimensional feature space – here representing crabs and lobsters.

### Implications for Memory Representation Models

The present findings can be explained in the framework of norm-based theories of face coding (Valentine, 1991, 2001) and shape coding (Suzuki, 2005). In the context of these theories, it has been proposed that individual faces might be represented by their deviations from an average face that is abstracted from encountered faces. This norm or average face is thought to serve as a reference point for all individual faces in the face space. Accordingly, it has been argued that the face adaptation procedure is likely to alter one or more multiple face norms along the manipulated feature dimension (e.g., age, gender, ethnicity, etc.) toward the adapting stimuli. This would result in a change of the distance between the adapted face norm and individual exemplars.

The current findings also demonstrate the contrastive nature of adaptation aftereffects previously seen in face and shape adaptation studies. That is, after presenting an adaptor (e.g., a lobster), test stimuli were categorized in the opposite direction (e.g., ratings were more crab-like). This finding is consistent with the assumption of a change of the distance between the adapted object norm and the individual exemplars. Thus, the natural zoological objects used in the present study may also be represented in a norm-based feature space.

Just as for faces (Rhodes et al., 2011) and shapes (Pasupathy and Connor, 2001; Kayaert et al., 2005), there are signs for separate norms in the space of crab and lobster categories in our Experiments 1 and 3. This assumption, presumably, explains the observation that test stimuli close to the continuum’s endpoints showed weaker adaptation aftereffects in contrast to test stimuli close to the midpoint. The weaker peripheral aftereffects could result from weaker effects of adaptor stimuli opposite the test stimuli. This observation is consistent with the assumption that there is not only one object norm, but more than a single norm abstracted from morphologically (as well as phylogenetically) related zoological objects such as crabs and lobsters.

Importantly, the probability of activating a norm corresponding to a crab or a lobster seems to increase under conditions in which the adaptor and the test stimuli provide a sufficient context or cue about the possible categorization of the presented objects as members of the crustacean category. This was the case in Experiment 1 in which we presented adaptor and test stimuli enriched with claws and legs and in which we found larger adaptation aftereffects as compared to Experiments 2 and 3. The fact that we found larger adaptation aftereffects in a condition with cues, i.e., claws and legs, in both the adaptor and the test stimuli, as compared to a condition with cues exclusively in the test stimuli, suggests that participants are activating the corresponding norm starting with the presentation of the adaptor stimulus. Interestingly, in the present experiments, the cue-enhanced adaptation aftereffects occurred despite the fact that the exact same set of claws and legs was used for the crab carapace and the lobster carapace, which excluded the possibility of recognizing the category by processing pure cue information, i.e., information from the claws and legs. Taken together, these findings suggest that in order to activate the respective norms, the cues need not to be specific for the specific category but rather activate the corresponding memory representation in a more general abstract way; otherwise the aftereffects are significantly smaller (cf. adaptation to the simple geometric shapes, as seen in Experiments 2 and 3).

The specific data pattern of the adaptation effects in Experiments 2 and 3 does allow us to discuss an alternative assumption, according to which the mere shape similarity between the adaptor and the test stimulus is decisive for the emergence of a full adaptation aftereffect. This could have been assumed on the basis of recent neuroimaging studies (Op de Beeck et al., 2008; see also Freud et al., 2015, 2017), which demonstrate the importance of shape similarity for object category processing in humans by analyzing brain activation data in similarity ratings. According to these findings, the relevance of outer shape similarity between objects for similarity judgments across objects increases across the early visual processing stages of object representation in the brain, which is especially pronounced in artificial object representations. However, at later processing stages of the visual processing hierarchy, the importance of outer shape similarity for the category representations of objects decreases; the latter pointing to an increasing contribution of additional feature representations of the presented objects allowing for higher-order object processing including familiarity decisions (Op de Beeck et al., 2008; Freud et al., 2017). These findings, in combination with the current results, allow for conclusions about the importance of the outer shape similarity between adaptor and test stimulus for the emergence of the full magnitude of the adaptation effects. In brief, the magnitude of the adaptation effect was similar between Experiment 2, with the same outer shape of adaptor and test stimulus, and Experiment 3, with a different outer shape of adaptor and test stimulus. Importantly, the adaptation effects in both conditions were smaller than those in Experiment 1, in which the presentation of additional information of claws and legs provided the context allowing for an enriched processing of the presented objects as members of the crustacean categories, and which led to increased adaptation effects in the latter condition.

Although further experimentation is required for a conclusive decision – perhaps by measuring brain activity data in the visual pathway – the current findings are consistent with the assumption that different stages in the visual processing hierarchy contribute to the full emergence of the adaptation aftereffects in complex objects; shape information seems to play an important role; and this is complemented by the processing of further features, beyond discriminative shape information, allowing for higher order processing of the presented objects as members of the crustacean decapod categories.

### Potential Confounds and Limitations

One potential limitation of the present study is the fact that we conducted a between-groups comparison where, instead of the differences across the experiments themselves, the results can hypothetically reflect inter-individual variation. However, we attempted to overcome the possible bias by creating homogenous groups and by having identical testing circumstances.

Another possible limitation of the present study could be that we did not completely control for the possible impact of adaptation effects occurring on retinal levels very early in the processing stream (see Webster, 2011; Webster and MacLeod, 2011). In more detail, for the case of investigations of face adaptation aftereffects, some studies have eliminated retinal adaptation by presenting adaptor and test stimuli at different display positions, with different sizes, and/or orientations (e.g., Zhao and Chubb, 2001; Carbon et al., 2007). However, while we did not introduce these particular differences, Experiment 3 allows us to at least speculate about the role of differences between adaptor and test stimuli, because this experiment used structurally dissimilar images during adaptation and test: we presented simple contour objects during adaptation and naturally complex objects during test, and were able to elicit adaptation aftereffects that were numerically stronger than in Experiment 2 with structurally identical adaptation and test images (i.e., both types were simple contour images). Interestingly, studies thoroughly controlling for retinotopic effects have also found similar patterns of results. Thus, we can be fairly confident that our effects are not pure retinal adaptation aftereffects.

Another minor yet possible limitation of our results might be the fact that the participants’ category ratings were slightly skewed and biased toward the “crab” side. Although a photograph of a genuine lobster was used to generate the lobster norm (which served as the lobster endpoint of the morphing as well as the 100% lobster adaptor in the experiment), the participants did not consider the test images with a higher percentage of lobster features to be very similar to the lobster images displayed during the learning phase. This is visible on the data plots (i.e., the ratings never reached the “lobster” end of the rating scale; **Figure 4**); and participants also reported this during the debriefing at the end of the experiment. We presume this bias of the used material to be due to the individual images created for the current experiment. That is, the “crab” stimulus was potentially more distinct than the “lobster” stimulus and, therefore, ratings rather tended toward to “crab” side.

A final potential criticism of our study relates to the specificity and generalizability of the material. Laboratory experiments hold their value when the results of individual paradigms and settings can be transferred to other settings, material, etc. To investigate adaptation aftereffects in complex natural object categories (different from the previously investigated category of faces), we first had to make sure that the relevant category representations of crustaceans were available to the participants by first familiarizing them, a process during which they presumably generated the respective norms. Since we used different material for the familiarization and the test phase (and did not show the experimental prototypes during familiarization), our results seem not to be restricted to the specific stimuli presented in the current experiment. Thus, we believe that we can generalize our findings on aftereffects to different exemplars of the crustacean category as well.

## Summary and Conclusion

The current results show that, similar to the results for human faces and simple shapes, object representations of animal categories are also subject to the influence of previous perceptual information resulting in adaptation aftereffects. Second, the complexity of this information determines the magnitude of the adaptation aftereffects. Third, the degree of the representations’ flexibility is proportional to their distance from the corresponding norms (i.e., the ones close to a prototypical norm are hard to influence and easy to categorize, while ambiguous ones away from and between prototypes are quite flexible yet difficult to classify). Fourth, adaptation to the objects’ parts can transfer to and result in aftereffects in the objects themselves. Taken together, these results indicate the high degree of the flexibility of the mental representations, as well as the processing of the representations of natural zoological objects such as those used in the present study. Both representations and processing appear to happen in a norm-based, multidimensional feature space. Further experimentation is needed to specify whether the representations of the decapods do indeed follow the principles of norm-based coding of presented objects during perception.

## Ethics Statement

All procedures performed in the study were in accordance with the standards of the Ethics Committee of the Humboldt-Universität zu Berlin and with the 1964 Helsinki Declaration and its later amendments. The Ethics Committee of the Humboldt-Universität zu Berlin approved the study. Written informed consent was obtained from all participants.

## Data Availability

The dataset containing all participants’ data is available under: https://zenodo.org/record/1228073.

## Author Contributions

AR, TiS, CB, GS, and ToS: conception of the experimental design and of data analysis, data interpretation, writing of the article. AR and TiS: conducting of the experimental investigation.

## Conflict of Interest Statement

The authors declare that the research was conducted in the absence of any commercial or financial relationships that could be construed as a potential conflict of interest. The reviewer B-SH and handling Editor declared their shared affiliation.

## Footnotes

1 Traditionally, the presence of a tail has been a major aspect in the classification of decapods. In the 19th century, decapods were subdivided into groups according to the characteristic of the hind body, namely the Macrura (literally: large/long tail), the Anomura (literally: irregular tail), and the Brachyura (literally: short tail). Although this classification is intuitive and still used by laypeople, in today’s phylogenetic systematics the Macrura are no valid group and the still accepted Anomura and Brachyura are combined in a group called Meiura (reduced tail; Scholtz and Richter, 1995). However, the tail represents an all-or-none feature for the category membership of exemplars, which would make its simultaneous manipulation with the carapace inconceivable. Besides, the presence/absence of the different tail parts of crabs and lobsters could strongly influence the subjective category ratings and, as a result, bias the effect of the adaptors. Therefore, we disregarded the tail during the creation of the experimental material (i.e., applied the morphing technique only on the carapaces) and covered the lower parts of the final stimuli, to put the study’s emphasis exclusively on the carapace and to measure purely the effect of the adaptors.

2 The novel nature of our experimental material and our interest in the participants’ subjective category judgments also lead us to choose a 5-point rating scale instead of the commonly used 2-alternative forced choice task, enabling us to more precisely measure the participants’ ratings and assess whether the objective midpoint of the morphing continuum coincides with their subjective midpoints.

3 To confirm these results, we also conducted a control analysis on the points of subjective equality (PSEs) reflecting those points on a presumed continuum between crab and lobster morphs, at which participants perceive a test image as the midpoint between crab and lobster. To that end, we fitted a Weibull curve (cf. Reindl et al., 2015) to participants’ category ratings at the seven morphing stages separately for the two adaptor conditions, calculated the PSEs from the fitted values, and compared the resulting PSEs in the crab and the lobster conditions with *t*-tests separately for each experiment. As a result, the comparisons of the estimated PSE values revealed significant differences in all three experiments (*t*s ≥ 2.354, *p*s ≤ 0.027). Though preliminary, these results emphasize the validity of our raw data–based conclusions on the emergence of adaptation effects in the current material. Note that a significant difference between the PSEs of the two conditions indicates that the presentation of an adaptor (i.e., of a crab adaptor) causes a shift in the processing of the morphed test images; in particular, it causes that an increased amount of morph information from the opposite test image (i.e., of the lobster) would be required to perceive the presented morphed image as at the midpoint between the crab and the lobster categories. Further studies are necessary to approve this and to investigate in more detail the precise amount of the shift in the PSE under different conditions of judgments and image complexity.
